# High Growth Rate of Diatoms Explained by Reduced Carbon Requirement and Low Energy Cost of Silica Deposition

**DOI:** 10.1128/spectrum.03311-22

**Published:** 2023-04-03

**Authors:** Keisuke Inomura, Juan José Pierella Karlusich, Stephanie Dutkiewicz, Curtis Deutsch, Paul J. Harrison, Chris Bowler

**Affiliations:** a Graduate School of Oceanography, University of Rhode Island, Narragansett, Rhode Island, USA; b Institut de Biologie de l'Ecole Normale Supérieure, CNRS, INSERM, Université Paris Sciences et Lettres, Paris, France; c Faculty of Arts and Sciences, Division of Science, Harvard University, Cambridge, Massachusetts, USA; d Department of Earth, Atmospheric, and Planetary Sciences, Massachusetts Institute of Technology, Cambridge, Massachusetts, USA; e Department of Geosciences, Princeton University, Princeton, New Jersey, USA; f Department of Earth, Ocean, and Atmospheric Sciences, University of British Columbia, Vancouver, British Columbia, Canada; University of Texas at San Antonio

**Keywords:** *Tara* Oceans, computer modeling, diatom, ecosystem, growth, microbiology, oceanography, photosynthesis, phytoplankton, silica frustule

## Abstract

The rapid growth of diatoms makes them one of the most pervasive and productive types of plankton in the world’s ocean, but the physiological basis for their high growth rates remains poorly understood. Here, we evaluate the factors that elevate diatom growth rates, relative to other plankton, using a steady-state metabolic flux model that computes the photosynthetic C source from intracellular light attenuation and the carbon cost of growth from empirical cell C quotas, across a wide range of cell sizes. For both diatoms and other phytoplankton, growth rates decline with increased cell volume, consistent with observations, because the C cost of division increases with size faster than photosynthesis. However, the model predicts overall higher growth rates for diatoms due to reduced C requirements and the low energetic cost of Si deposition. The C savings from the silica frustule are supported by metatranscriptomic data from *Tara* Oceans, which show that the abundance of transcripts for cytoskeleton components in diatoms is lower than in other phytoplankton. Our results highlight the importance of understanding the origins of phylogenetic differences in cellular C quotas, and suggest that the evolution of silica frustules may play a critical role in the global dominance of marine diatoms.

**IMPORTANCE** This study addresses a longstanding issue regarding diatoms, namely, their fast growth. Diatoms, which broadly are phytoplankton with silica frustules, are the world’s most productive microorganisms and dominate in polar and upwelling regions. Their dominance is largely supported by their high growth rate, but the physiological reasoning behind that characteristic has been obscure. In this study, we combine a quantitative model and metatranscriptomic approaches and show that diatoms' low carbon requirements and low energy costs for silica frustule production are the key factors supporting their fast growth. Our study suggests that the effective use of energy-efficient silica as a cellular structure, instead of carbon, enables diatoms to be the most productive organisms in the global ocean.

## INTRODUCTION

**FIG 1:**
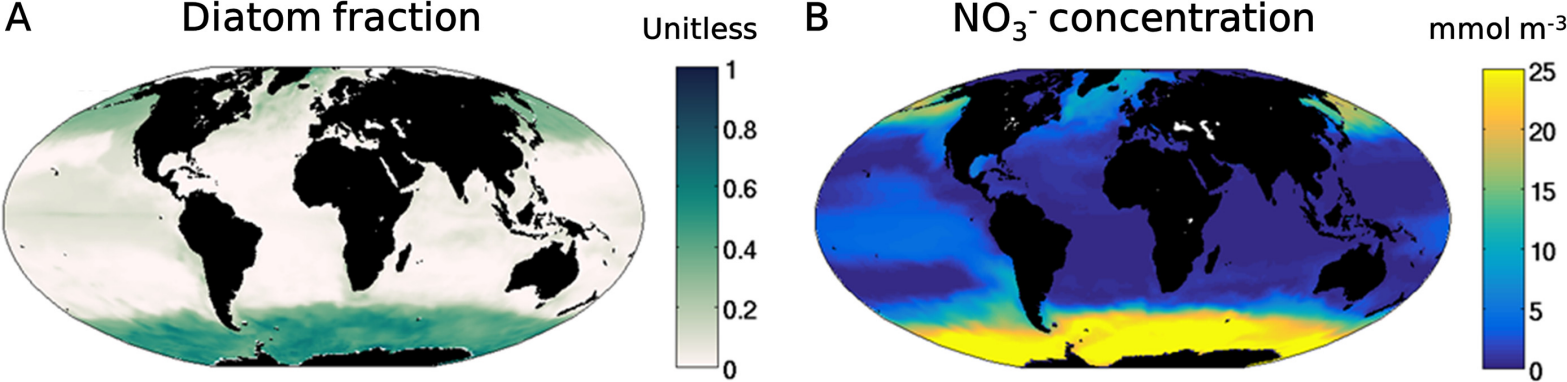
Recent simulations of the fraction of global diatom biomass and nitrate concentrations. The fraction of diatom biomass (A) and NO_3_^−^ concentrations (B) were simulated with the marine ecological model MITgcm (19). These plots show that the annual means averaged between 0 m and 50 m in depth. The general distribution patterns are consistent with observations (see Fig. S6 in reference 19).

Diatoms are major photosynthesizers in the ocean, accounting for up to 45% of marine primary production, more than that of all the world’s tropical rainforests (1). Diatoms are uniquely characterized by silica (SiO_2_) frustules (shell) (2–7) and display a diverse set of molecular responses to environmental conditions (8–10). They are major components of the ecosystem at high latitudes and in coastal regions where nutrients are abundant (11–13). Such dominance requires high growth rates, which enable populations to proliferate rapidly under conditions with high nutrient levels (14–16). The global niches of diatoms can be captured in marine ecosystem models with the assumption that they have the highest maximum growth rate among the phytoplankton, with a trade-off of a high half-saturation constant (17–20) (output from the model in reference 19 is shown in Fig. 1). The high growth rate in combination with lower palatability allows diatom dominance in environments with high/fluctuating levels of nutrients, whereas other plankton may have advantages in environments with stable low levels of nutrients, where high nutrient affinities are favored (19).

**FIG 2:**
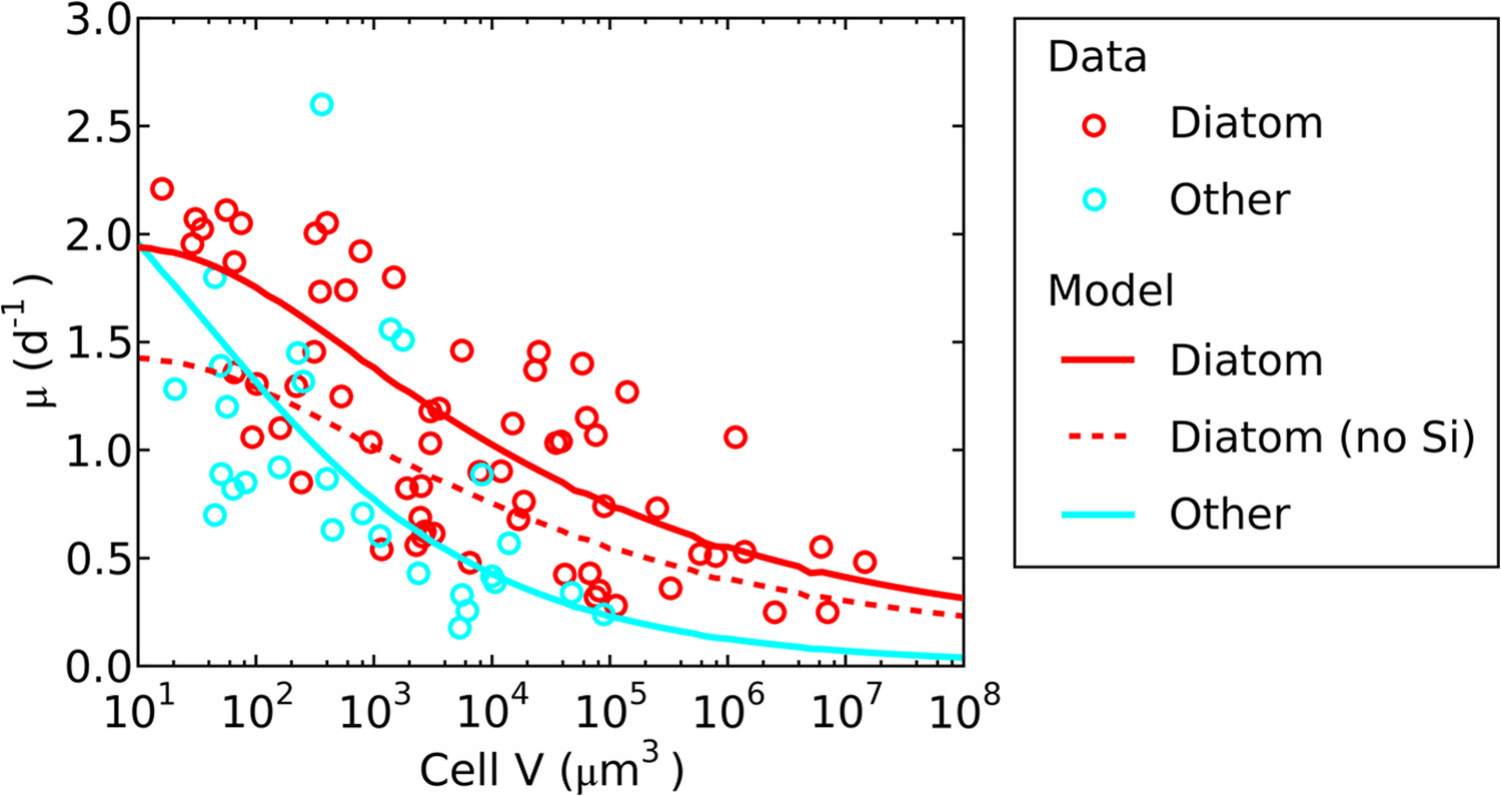
Model data comparison of nutrient-replete growth rate (μ)-cell volume (*V*) relationships. Data are from reference 23. Other includes chlorophytes, dinoflagellates, coccolithophores, haptophytes, and raphidophytes. Diatom (no Si) indicates the simulation with the C cost of non-silica biomass production applied to silica frustule production.

High maximum growth rates for diatoms assumed by ecosystem models are largely consistent with laboratory observations of growth rates under nutrient-replete conditions, despite considerable variations (21–23) (Fig. 2, circles). However, the causes of such high diatom growth rates remain elusive. It has been suggested that the production of the silica frustule is energetically efficient (24, 25), yet such an effect has not been quantitatively analyzed at a cellular scale. How does energy efficiency influence the growth of diatoms? Diatoms have also been found to have a low C (carbon) per volume, relative to other phytoplankton, e.g., a compilation of cellular C-volume relationships shows that diatoms display a lower C per specific volume than other phytoplankton (26), suggesting that the C cost of reproduction for a given cell size should be lower for diatoms. How does this pattern influence the growth rate?

**FIG 3:**
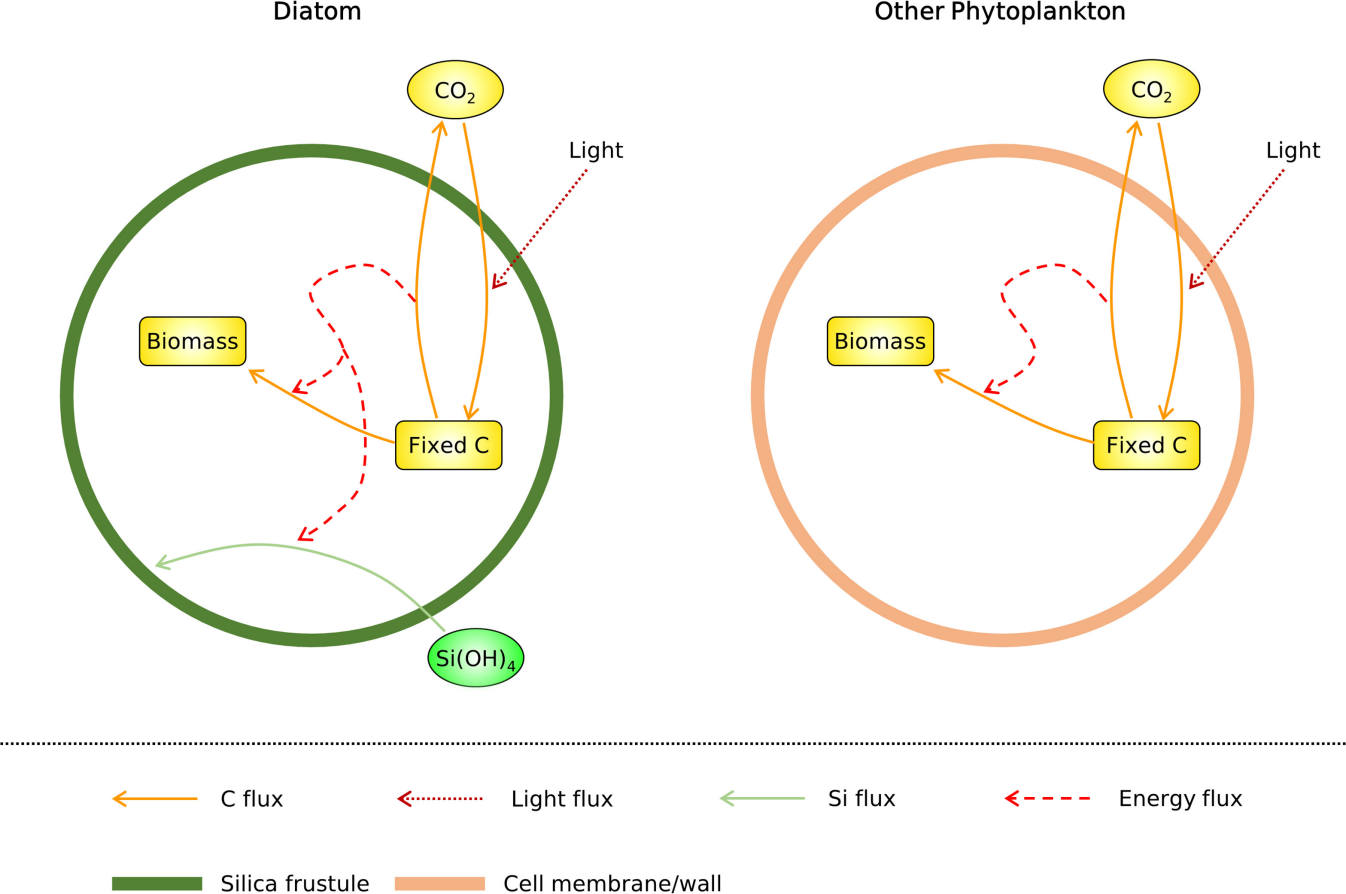
Schematics of the coarse-grained models of diatoms (CFM-Diatom) and non-diatom phytoplankton. Here, biomass represents all of the final cellular material that contains carbon.

In addition to the high growth rate of diatoms, there is a clear trend between cell volumes and growth rates. Above the cell volume of 10^2^ μm^3^, the growth rates of diatoms and other phytoplankton decrease (Fig. 2) (23, 27–30). Multiple theories have been proposed for this trend. First, increasing cell size increases intracellular distances (for example, between organelles), thus decreasing the speed of molecular transport and processing and decreasing the growth rate (30, 31). Second, increasing cell size increases the packaging effect (intracellular light absorption), thus decreasing the photosynthesis rate per volume and decreasing the growth rate (30, 32). To test this effect in the context of whole-cell metabolism, here we develop a model (Cell Flux model of a Diatom [CFM-Diatom]) (Fig. 3) by combining a simple model of light absorption within a cell with a cellular model of simple metabolism (33–35) to analyze the general trends in nutrient-replete growth rates across orders of magnitude of cell size.

## RESULTS AND DISCUSSION

### Model prediction of the growth rates.

CFM-Diatom is a simple metabolic flux model that resolves essential metabolism, including C fixation (photosynthesis), respiration, generation of biomass, and silica deposition (Fig. 3). These fluxes are constrained by mass, electron, and energy balances under a steady-state assumption. The model also resolves the effect of intracellular light attenuation. The model differentiates diatoms from other phytoplankton on the basis of the existence of silica deposition and cellular C per volume (details in Materials and Methods). Despite its simplicity, the model captures the general trend of growth rate-cell volume relationships for both diatoms and other phytoplankton (Fig. 2). The growth rates of diatoms and other phytoplankton decrease with increased cell volume, but diatoms have higher growth rates at a specific volume, consistent with the compiled data (Fig. 2).

### Intracellular light attenuation results in lower growth rates with larger cell volumes.

**FIG 4:**
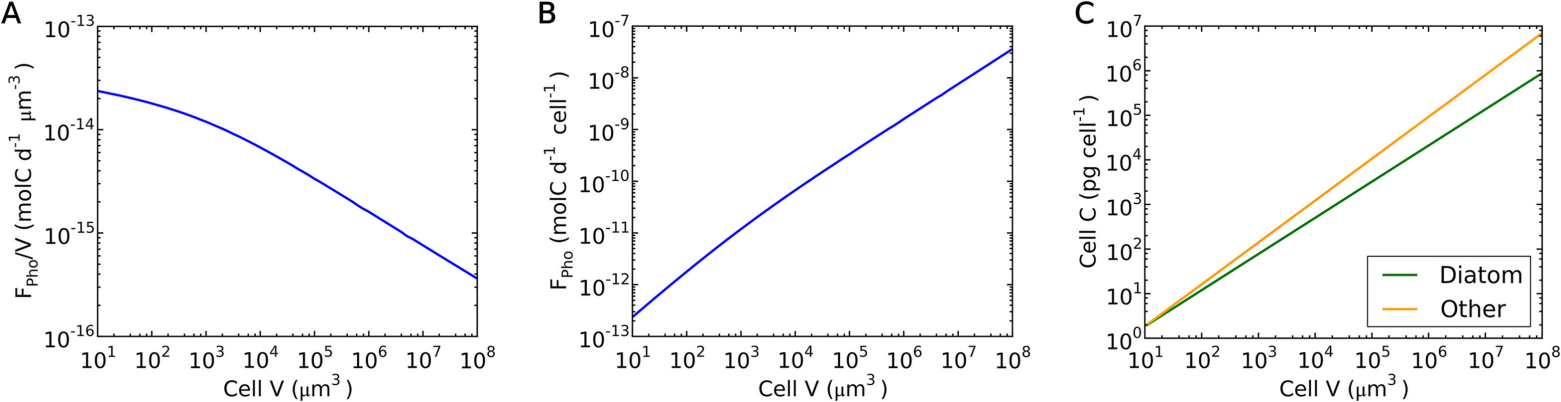
Simulated photosynthesis rates for different cell volumes and observed relationships between cellular C and cell volume. (A) Simulated photosynthesis rate (per volume)-volume relationship. (B) Simulated photosynthesis rate (per cell)-volume relationship. (C) Cellular C-volume relationship of diatoms and other phytoplankton (26).

The general trend of decreasing growth rate with increasing cell volume is predicted as a consequence of intracellular light attenuation (Fig. 2 and 4). As the cell volume increases, the path length of light increases within the cell, reducing light intensity per volume of the cell. This effect leads to a lower per-volume rate of photosynthesis (Fig. 4A) and a decreased slope of the cellular photosynthesis rate-volume relationship (Fig. 4B). This decreased slope, in turn, results in decreased growth rates with increased cell volume (Fig. 2), since the cellular C does not decrease as severely with increased volume (Fig. 4C), and the growth rate is proportional to the photosynthesis rate per cellular C (*F*_*Pho*_/*Q*_*C*_).

### Low C requirement and low energetic cost of silica deposition support high growth rates of diatoms.

**FIG 5:**
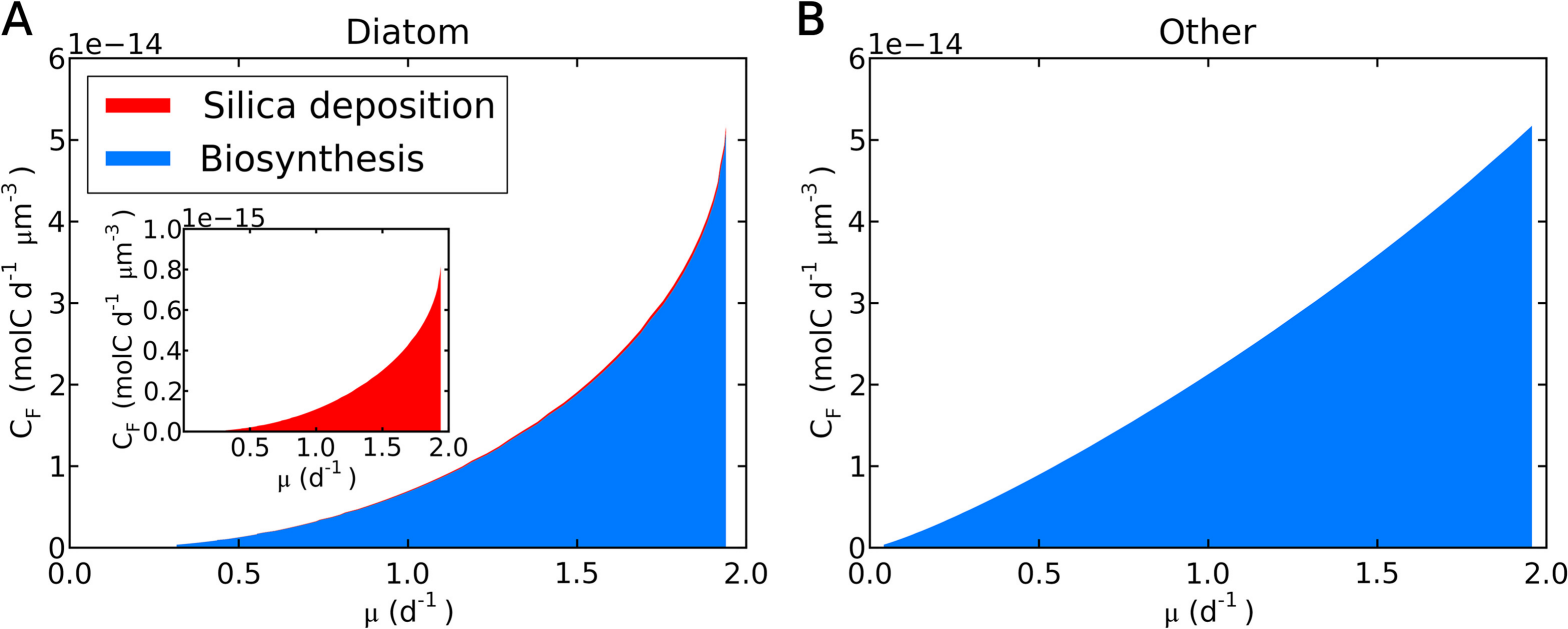
Simulated fate of C (C_F_) for various growth rates under nutrient repletion. (A) Diatoms. (B) Other phytoplankton of the same size. The *y* axes indicate C flux per cell volume. In panel A, there is a slight layer in red (C cost for silica deposition) on top of the blue (C cost for biosynthesis); the C for silica deposition is almost invisibly small. To make it visible, the inset shows C cost for silica deposition with a different range on the *y* axis.

What explains the differences in growth rate between diatoms and other phytoplankton? Our model predicts the observed difference (Fig. 2), based on the reduced C requirement per cell (Fig. 4C) and the extremely low cost of silica deposition (Fig. 5). The reduced C requirement is based on the compilation of *Q*_*C*_ versus size for different taxa of phytoplankton (26). It has been shown that diatoms follow a distinct curve, compared with other taxa, with lower *Q*_*C*_ values (Fig. 4C). We applied this relationship in the model with the observed different values for *A*_C_ and *B*_C_ (26) and reproduced the observed trend by using the same rate of photosynthesis for diatoms and non-diatoms. The predicted growth rate of diatoms due to the higher specific rates of photosynthesis (photosynthesis rate per C) are because of the lower cellular C; this is qualitatively supported by the observation of a relatively high content of chlorophyll per C in diatoms (36). Other physiological factors could potentially contribute to high specific photosynthesis rates of diatoms, such as carbon concentration mechanisms (37–39), chloroplast-mitochondrion coupling (40), and the use of silica frustules for enhanced light harvesting (41).

Although the C requirement for diatoms is small, there is a cost specific to diatoms, i.e., silica deposition. Although the cost for silica deposition per Si (silicon) has been estimated (24, 25), the silica deposition cost relative to the entire C cost has not been quantified. Our model shows that the C cost for silica deposition is minimal, accounting for only ~1.6% of the entire carbon cost (Fig. 5A), which includes C for biosynthesis and C for respiration to support biosynthesis. This prediction of a low cost of Si is partially due to the low C cost per Si (0.167 mol C mol Si^−1^), compared to the C costs per biomass C production (1 + *E*_μ_) (1.691 mol C mol C^−1^), and partially due to the relative amounts of Si versus C (here, 0.163 [maximum value from reference 42]) (see Table S2 in the supplemental material). Despite the application of a Si/C ratio at the high end of the observed range (42), the total energy cost of silica deposition is small, suggesting that the energetic effect of variations in Si/C is negligible on a cellular scale. Although the cells could have even higher Si content, especially with excess Si availability (43, 44), the results of total C cost and growth rate would not change significantly (e.g., see Fig. S2) since the silica deposition cost is extremely small. On the other hand, if we apply the C cost of non-silica biomass production to the production of silica frustule, the model predicts substantially lowered growth rate (Fig. 2; compare dashed and solid red curves), underpinning the importance of low Si cost.

### Hypothesis arising from this study: Si replacement of C in structural material.

The model results show that the reduced C requirement and low energetic costs could be key factors underlying the high growth rate of diatoms. This leads us to hypothesize that, quantitatively, Si takes over some functional roles of C, reducing C demand and energetic costs and leading to a high growth rate. For example, cells need proteins to maintain their structure (e.g., cytoskeleton) (45, 46). Also, phytoplankton typically have cell walls that support cell morphology (47–49). These C-rich structural molecules may be reduced in diatoms, since their silica frustules provide the rigid support for maintaining cell structure. While diatoms may require additional resources to construct their silica frustules, we predict that the resource requirement is minimal because the template contains only one layer of amino acid residues (2).

Although silica frustules may provide structural support, the frustules cannot replace molecules that are related to growth, since they do not generally facilitate biochemical processes as enzymes do. For example, they cannot replace photosystems and enzymes for C fixation. As a result, the C-specific abundance of these synthesis-facilitating molecules increases as the C-rich structure is replaced by the silica frustules. We point out that vacuoles may be one of the causes of low C content (26), which would reduce the intracellular space for photosynthetic molecules. Also, it is possible that vacuoles are rather the outcome of reduced structural material.

### Exploring the hypothesis with *Tara* Oceans data: diatoms invest high transcriptional efforts into protein synthesis but not into cytoskeleton maintenance.

**FIG 6:**
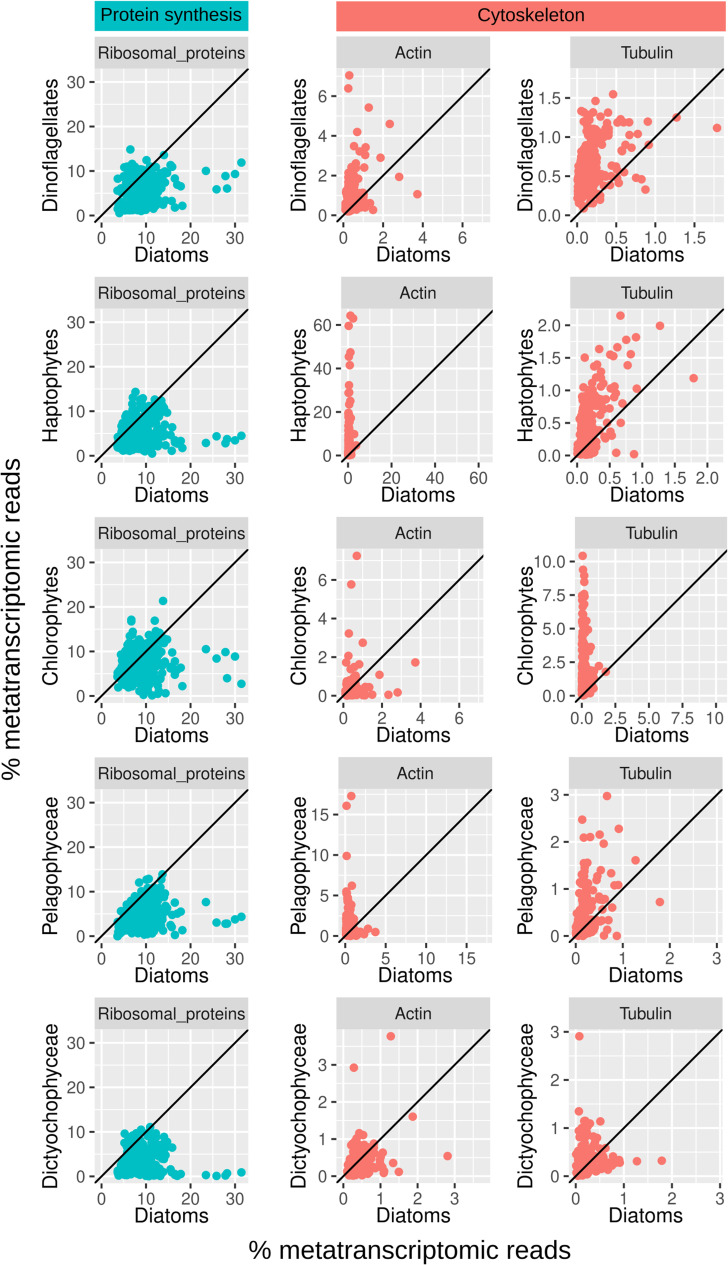
Transcriptional efforts to maintain proteins involved in protein synthesis (ribosomal proteins) and the cytoskeleton (actin and tubulin) in marine environmental populations of diatoms versus other photosynthetic protists. The analyzed data correspond to the metatranscriptomes generated by *Tara* Oceans across the main ocean regions (see Fig. S1 in the supplemental material) (52, 53). The scatterplots compare the relative abundances (proportion of metatranscriptomic reads for the given function among the total metatranscriptomic reads from the corresponding taxon) between diatoms and other photosynthetic protists. Each point corresponds to a size-fractionated seawater sample. Axes are on the same scale, and the diagonal lines correspond to a 1:1 slope.

At steady state, the growth rate of phytoplankton depends on the protein synthesis capacity of the cell and thus on the number of active ribosomes (50, 51). Here, we examined the metatranscriptomes from samples collected by *Tara* Oceans from the main ocean basins (52, 53) (Fig. 6) to determine the proportion of transcripts coding for ribosomal proteins among the different phytoplankton taxa. Our results showed that diatoms generally have greater relative abundances of transcripts for ribosomal proteins than do the other phytoplankton groups (Fig. 6), possibly reflecting their higher growth rates due to the replacement of some cellular C by Si.

In addition, the rigid support of the silica frustule can reduce diatom efforts in maintaining their structure using the cytoskeleton. We examined this possibility by analyzing the relative abundances of transcripts coding for the main cytoskeleton components, actin and tubulin, and found that diatoms indeed have lower levels of transcripts for them, in comparison with other phytoplankton groups (Fig. 6).

Our model focuses on the general trend of the growth rates for diatoms versus nondiatoms. Thus, it mainly focuses on the averaged pattern. However, there are significant variations even within a diatom group. Morphologically, some are chain-forming and others are solitary. Some are found in freshwater, while others are found in marine environments. Even within the ocean, some exist in coastal environments, while others are found in the open ocean. Furthermore, different diatoms contribute differently to the biological carbon pump (54). Possibly due to these differences, our data show considerable variations. Despite this, our results highlight two factors that could generate variation in diatom growth rates even within a given size class. First, diatoms exhibit high variability in *Q*_C_ at each size, and this would translate directly into growth rate differences. Second, the degree of silicification can vary among species and according to other growth conditions. Further studies are needed to identify and incorporate additional factors into the model to improve its performance. One of these factors is likely to be iron, which is known to impact diatom growth strongly, as well as to influence frustule thickness (55–57). Additional uncertainty may involve the production of extracellular polysaccharides (58), which may reduce the overall growth rate because it contributes to the loss of C (see Fig. S3). However, such C excretion is not exclusive to diatoms (59–62), and how it affects the relative growth rate has not been clear. For example, if diatoms have lower C excretion than other phytoplankton, this may contribute to a higher growth rate of diatoms, but such a trend has not been well supported so far.

### Conclusions.

**FIG 7:**
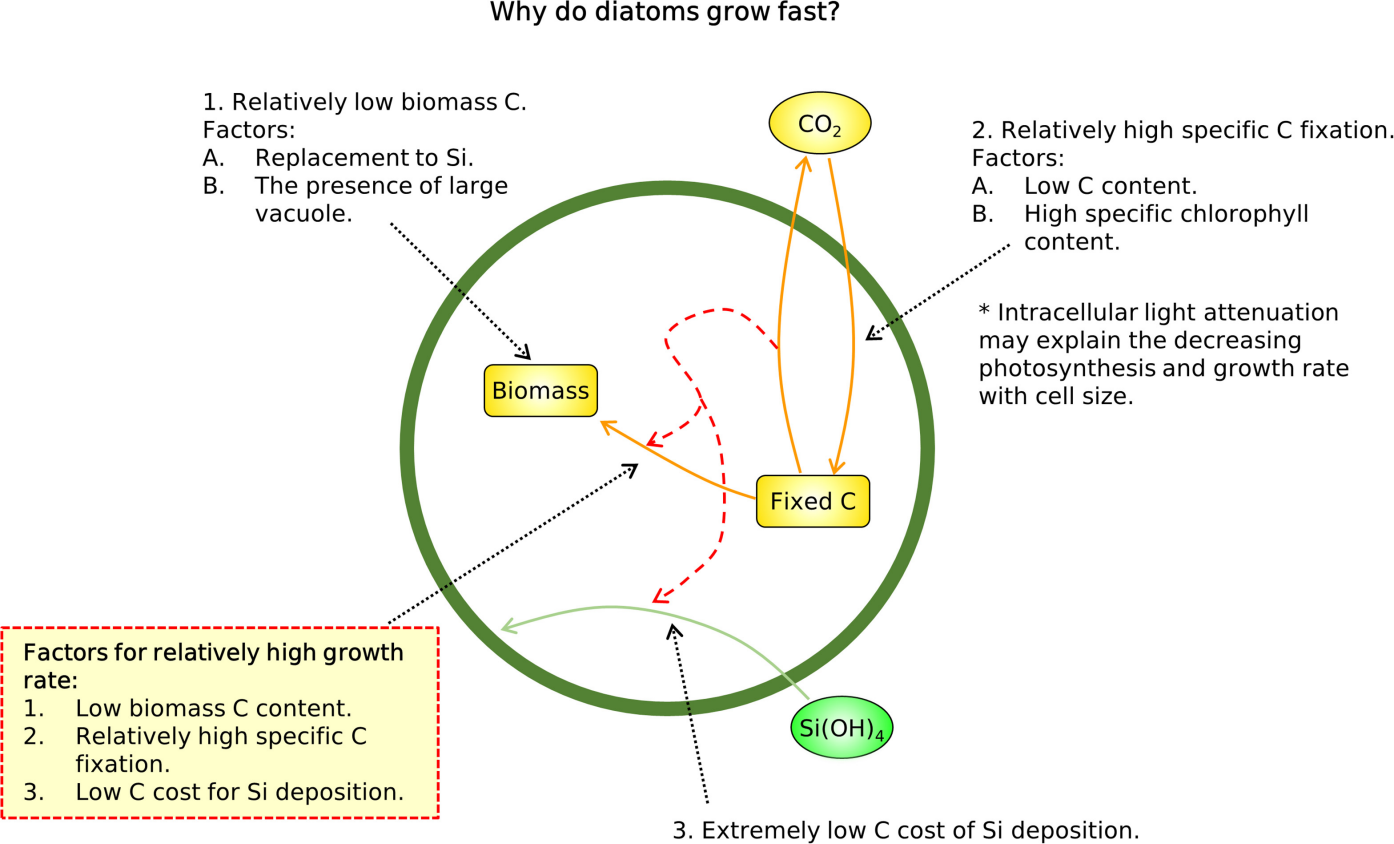
Schematic summary of this study. Key conclusions are within the dashed red frame.

To investigate what causes high growth rates of diatoms under nutrient repletion, we have developed a simple metabolic model of diatoms (CFM-Diatom), focusing on C metabolism. The model captures the observed high maximum growth rates of diatoms relative to other phytoplankton, based on relatively low requirements for C for cellular material, resulting in high specific C fixation rates and an extremely low energetic cost for Si deposition (Fig. 7). These results led us to hypothesize that structural material, which is rich in C, is replaced by silica frustules, reducing cellular C requirements for growth and enriching growth-related molecules, such as those for light harvesting and C fixation, as well as for various molecular machineries (Fig. 7). This hypothesis is consistent with an interpretation of *Tara* Oceans data, which show that the transcript abundance for cytoskeleton components in diatoms is lower than in other phytoplankton, suggesting that the rigid support of the silica frustule can reduce the energy costs for maintaining their structure; the diatoms can then channel resources into growth, as reflected by the greater transcript abundance for ribosomal proteins. Our simple model focusing on metabolism may be useful for investigating growth rate-volume relationships for other specific types of phytoplankton, such as calcifiers, diazotrophs, and cyanobacteria. CFM-Diatom may provide a mechanistic framework to predict the growth differences in ecosystem models, instead of relying on simple parameterizations. Finally, our study indicates the effective use of Si as a cellular structure instead of C, enabling diatoms to be the most productive primary producers in the global ocean.

## MATERIALS AND METHODS

To study why diatoms may grow quickly and why their growth decreases with increased volume, we have developed CFM-Diatom (Fig. 3). The model represents simple coarse-grained fluxes based on mass, energy, and electron budgets and is useful for providing quantitative information on a cellular scale (33–35, 63, 64). The model’s flexibility, computational efficiency, transparency, and ability to provide an intuitive whole-cell view complements more detailed models such as flux balance analysis (65–68). Here, to represent nutrient-replete situations (69), we focus on C fluxes in the model and test the effect of reduced C requirements in diatoms. We also simulate intracellular light attenuation and test the effect of volume on the growth rate. Furthermore, we explicitly represent silica deposition and quantify its C costs at a cellular scale. Finally, the importance of C savings by silica frustules is tested with *in situ* molecular data from environmental phytoplankton populations observed by the *Tara* Oceans expedition.

### CFM-Diatom.

Here, we describe the core equations of CFM-Diatom (Fig. 3). The parameter units and values are listed in Table S1 and S2 in the supplemental material. Since our focus is the maximum growth rate, we assume nutrient-saturated conditions (i.e., nitrogen, phosphorus, silicon, and iron are not limited). Under such conditions, the growth would be controlled by the rate of photosynthesis (or C fixation) (35, 69) and temperature (70, 71); here, we focus on the former and thus the C balance within the cell:

(1) $$\frac{dQ_{C}}{\mathrm{dt}}=F_{\mathrm{Pho}}-\mu Q_{C}\left(1+E_{\mu }+E_{\mathrm{Si}}\right)$$

where *Q*_*C*_ is the cellular C quota, *t* is time, *F*_*Pho*_ is the photosynthesis rate per cell, μ is the growth rate, *E*_μ_ is the growth respiration factor, and *E*_*Si*_ is a factor for silica deposition cost (24, 25). Assuming a steady state, we obtain a simple relation for growth rate:

(2) $$\mu =\frac{F_{\mathrm{Pho}}}{Q_{C}\left(1+E_{\mu }+E_{\mathrm{Si}}\right)}$$

Here, *Q*_*C*_ is calculated from a cell volume:

(3) $$Q_{C}=A_{C}V^{B_{C}}$$

where *A*_*C*_ and *B*_*C*_ are constant factors (26) and *V* is cellular volume. We differentiate diatom cells and other phytoplankton cells with two factors. First, *E*_*Si*_ applies only to diatom cells, because the creation of silica frustules is specific to diatom cells. Second, we applied different *A*_*C*_ and *B*_*C*_ values for diatoms and other cells (see the supplemental material), since diatoms show a distinct *Q*_*C*_-*V* pattern, with lower *Q*_*C*_ values than other phytoplankton (26). To obtain *F*_*Pho*_, we considered the effect of light attenuation within the cell following Beer’s Law (72) and a saturating curve of photosynthesis with light (see the supplemental material), which yields a decreased specific photosynthesis rate with size (Fig. 4A). Given the broad quantitative success of the simple model (Fig. 2), we hypothesize that the size-dependent light absorption coefficients have next-order impacts on the growth rate. Further experiments are needed to constrain such factors.

### Analysis of *Tara* Oceans metatranscriptomes.

*Tara* Oceans expeditions performed a worldwide sampling of plankton in the upper layers of the ocean between 2009 and 2013, covering the main ocean basins. A low-shear, nonintrusive, peristaltic pump and plankton nets of various mesh sizes were used on board *Tara* to sample and concentrate appropriate volumes of seawater to assess local eukaryotic biodiversity in four major organism size fractions, i.e., piconanoplankton (0.8 to 5 μm or 0.8 to 2,000 μm), nanoplankton (5 to 20 μm or 3 to 20 μm), microplankton (20 to 180 μm), and mesoplankton (180 to 2,000 μm) (see Fig. S1 of reference 53). Metatranscriptomes were generated from poly(A)^+^ RNA extracted from these samples (52, 53). The metatranscriptomic reads were assembled and then clustered at 95% identity. We searched this catalog for sequences coding for ribosomal proteins (112 Pfam models listed in Table S1 of reference 38) and for the cytoskeleton components actin (Pfam entry code PF00022) and tubulin (Pfam entry code PF00091) by running HMMer (version 3.2.1 with the gathering threshold option) over the translated sequences. Taxonomic assignment was carried out by sequence similarity against a reference database (52). Based on this assignment, we retained only sequences assigned to the main phytoplankton groups (note that we were not able to discard heterotrophic species from dinoflagellates due to the small number of reference sequences for this group). We finally retrieved the metatranscriptomic read abundances of the selected sequences and normalized them to the total read abundance for transcripts of the corresponding phytoplankton taxon in each sample. The resulting relative abundance values were compared between diatoms and other photosynthetic protists to infer the differences in the transcriptional efforts to maintain protein synthesis and the cytoskeleton. We focused the analysis on the surface samples (5-m depth) from the 66 stations (see Fig. S1) for which metatranscriptomic data are available.

### Data availability.

CFM-Diatom is written in Python 3.2.5 and is freely available from Zenodo (https://doi.org/10.5281/zenodo.3750213).
