# Supplementary material for: High Growth Rate of Diatoms Explained by Reduced Carbon Requirement and Low Energy Cost of Silica Deposition
Source: Microbiol Spectr. 2023 Apr 3;11(3):e03311-22. doi: 10.1128/spectrum.03311-22 (PMC10269801; doi:10.1128/spectrum.03311-22)
Supplement: Supplemental file 1 — Supplemental material. Download spectrum.03311-22-s0001.pdf, PDF file, 0.9 MB [file spectrum.03311-22-s0001.pdf]

Supplementary Material

Contents:

Supplementary Methods

Fig. S1, S2, S3

Tables S1, S2

Supplementary References

Supplementary Methods

Modeling light attenuation within the cell

To obtain F_{Pho}, we consider the effect of light attenuation within the cell, which is described by the following equation based on Beers' Law (1):

$$I = I_0 \, e^{-k_{Chl} \, Chl \, Z} \qquad \text{[eq. S1]}$$

where I is local light intensity, I_0 is source light intensity, k_{Chl} is extinction coefficient per chlorophyll, Chl is chlorophyll concentration and Z is the path length within the cell. We assumed the same chlorophyll content per cell across taxa as represented by the following equation:

$$Chl = \frac{Y_{Chl} \, Q_C^{Non-diatom}}{V} \qquad \text{[eq. S2]}$$

where Y_{Chl} is a chlorophyll conversion factor and $Q_C^{Non-diatom}$ is a cellular C quota of non-diatoms. We consider the spherical cellular shape and uniform distribution of chlorophyll within the cell. Once we obtain I at each point within the cell, we obtain local photosynthesis rate within the cell following a commonly used saturating relationship (2–4):

$$F_{Pho}^{Chl} = F_{Pho,max}^{Chl}(1 - e^{-A_I \, I}) \qquad \text{[eq. S3]}$$

where F_{Pho}^{Chl} is photosynthesis rate per chlorophyll, $F_{Pho,max}^{Chl}$ is the maximum photosynthesis rate per chlorophyll, A_I is a light harvesting coefficient, representing the combination of handling time and absorption cross-section. Then, we integrate the local rate of photosynthesis to the cellular level:

$$F_{Pho} = \int_V F_{Pho}^{Chl}\, Chl\, dv \qquad \text{[eq. S4]}$$

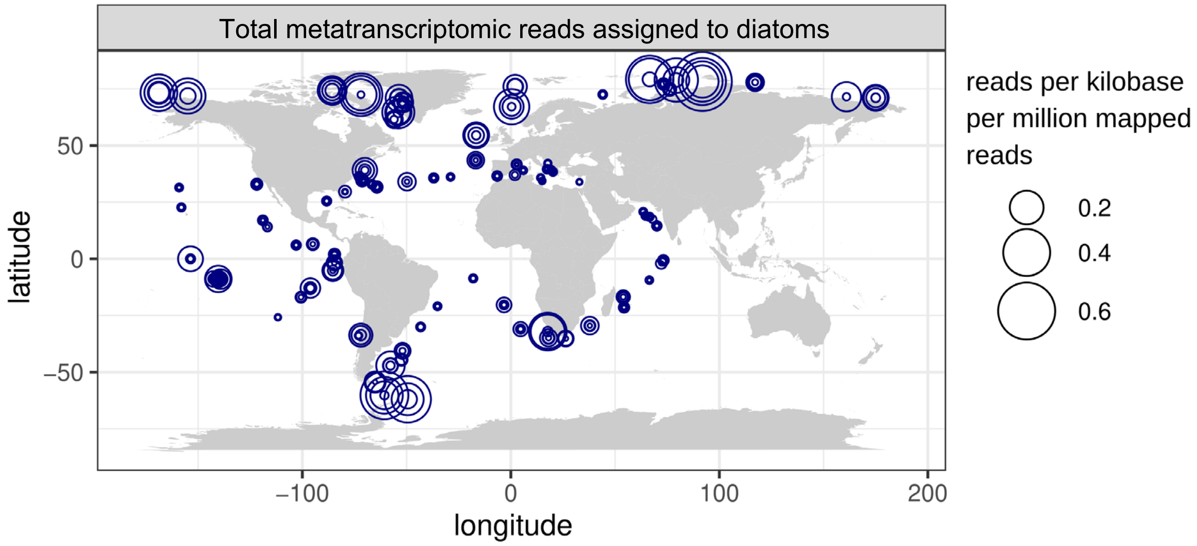

Fig. S1 Locations of *Tara* Oceans stations and total metatranscriptomic reads assigned to diatoms (5, 6).

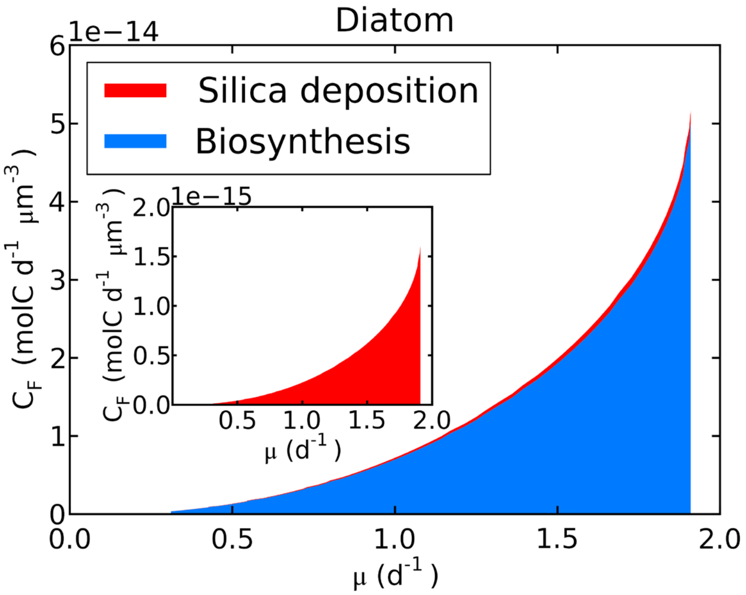

Fig. S2 Simulated fate of C (C_F) for various growth rates under nutrient repletion for diatom with doubled Si composition. The inset shows C cost for silica deposition in a different y axis range to make it visible.

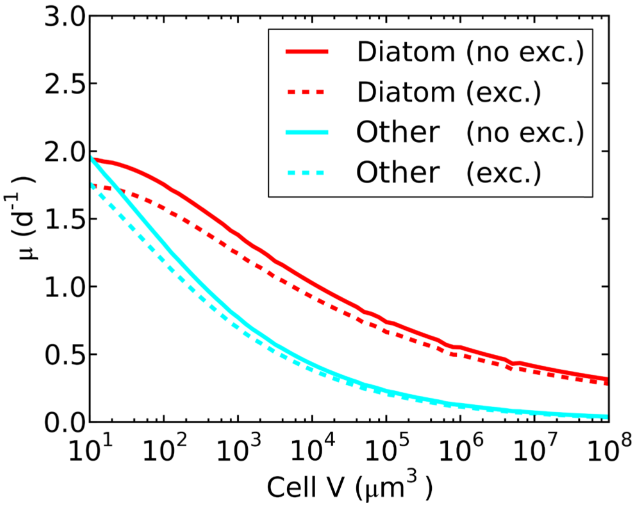

Fig. S3 Simulated effect of C excretion on nutrient replete growth rate (μ) – cell volume (V) relationship. The solid curves show the default simulation (as in Fig. 2) and the dashed curves show the simulation with C excretion (here 10% of total fixed C).

Table S1 Parameters, units and definitions

Parameter	Definition	Unit
Q_C	Cellular C quota	mol C $cell^{-1}$
t	time	t
F_{Pho}	C fixation rate per cell	mol C $cell^{-1}$ d^{-1}
μ	Growth rate	d^{-1}
E_μ	Growth cost factor	-
E_{Si}	Si accumulation cost factor	-
A_C	Cellular C quota factor	mol C μm^3
V	Cellular volume	μm^{-3} $cell^{-1}$
B_C	Cellular C quota power factor	-
I	Local light intensity	μmol m^{-2} s^{-1}
I_0	Source light intensity	μmol m^{-2} s^{-1}
k_{Chl}	Extinction coefficient per chlorophyll	μm^2 (mol C in $chlorophyll)^{-1}$
Chl	Chlorophyll concentration	(mol C in chlorophyll) μm^{-3}
Y_{Chl}	Chlorophyll conversion factor	(mol C in chlorophyll) mol C^{-1}
$Q_C^{Non-diatom}$	Cellular C quota of non-diatoms	mol C $cell^{-1}$
Z	Path length	μm
F_{Pho}^{Chl}	C fixation rate per chlorophyll	mol C d^{-1} (mol C in $chlorophyll)^{-1}$
$F_{Pho,max}^{Chl}$	Maximum C fixation rate per chlorophyll	mol C d^{-1} (mol C in $chlorophyll)^{-1}$
A_I	Light harvesting coefficient	μmol^{-1} m^2 s

-: dimensionless

Table S2 Parameter values for diatoms

Parameter	Value	Unit
Common values		
E_{μ}	[*1] 6.91×10^{-1}	-
k_{Chl}	4.90×10^{-9}	μm^2 (mol C in chlorophyll)$^{-1}$
I_0	[*2] 200	$\mu mol\ m^{-2}\ s^{-1}$
Y_{Chl}	[*3] 1.18×10^{-2}	(mol C in chlorophyll) mol C^{-1}
$F_{Pho,max}^{Chl}$	5.49×10^{2}	mol C d^{-1} (mol C in chlorophyll)$^{-1}$
A_I	[*4] 8.63×10^{-3}	$\mu mol^{-1}\ m^2\ s$
Diatom specific values		
A_C	[*5] 2.40×10^{-14}	mol C μm^3
B_C	[*5] 8.11×10^{-1}	-
E_{Si}	[*6] 2.72×10^{-2}	-
Non-diatom specific values		
A_C	[*5] 1.80×10^{-14}	mol C μm^3
B_C	[*5] 9.39×10^{-1}	-
E_{Si}	0.00	-

[*1] Estimated with mass, electron and energy balance (7) with the suggested energy transfer efficiency of 0.6 and stoichiometry of $C_5H_7O_2N_{0.75}$ (note: Redfield C:N (8)) with NO_3^- as N source.

[*2] Typical light intensity that gives maximum growth rate for diatoms and other phytoplankton (9).

[3*] Typical Chl:C ratio of non-diatoms (10, 11).

[4*] Value from (4).

[*5] Value based on (12).

[*6] Product of molar Si:C ratio of 1.63×10^{-1} (maximum value from (13)) and C cost per Si uptake of 1.67×10^{-1} (mol C mol Si^{-1}) (14). For the dashed curve in Fig. 2, this value (E_{Si}) is replaced by 6.45×10^{-1}, which represents the equivalent cost for biomass production (1+E_{μ}) for the same weight of C as Si.

Supplementary References

1. Liou KN. 2002. An Introduction to Atmospheric Radiation, International Geophysics Series, 2nd edn. Vol. 84. Academic Press, San Diego, CA, 1st ed. Academic Press.
2. Geider RJ, Macintyre HL, Kana TM. 1998. A dynamic regulatory model of phytoplanktonic acclimation to light, nutrients, and temperature. Limnology and Oceanography 43:679–694.
3. Cullen JJ. 1990. On models of growth and photosynthesis in phytoplankton. Deep-Sea Research 37:667–683.
4. Inomura K, Omta AW, Talmy D, Bragg J, Deutsch C, Follows MJ. 2020. A mechanistic model of macromolecular allocation, elemental stoichiometry, and growth rate in phytoplankton. Frontiers in Microbiology 11:86.
5. Carradec Q, Pelletier E, Da Silva C, Alberti A, Seeleuthner Y, Blanc-Mathieu R, Lima-Mendez G, Rocha F, Tirichine L, Labadie K, Kirilovsky A, Bertrand A, Engelen S, Madoui MA, Méheust R, Poulain J, Romac S, Richter DJ, Yoshikawa G, Dimier C, Kandels-Lewis S, Picheral M, Searson S, Acinas SG, Boss E, Follows M, Gorsky G, Grimsley N, Karp-Boss L, Krzic U, Pesant S, Reynaud EG, Sardet C, Sieracki M, Speich S, Stemmann L, Velayoudon D, Weissenbach J, Jaillon O, Aury JM, Karsenti E, Sullivan MB, Sunagawa S, Bork P, Not F, Hingamp P, Raes J, Guidi L, Ogata H, De Vargas C, Iudicone D, Bowler C, Wincker P. 2018. A global ocean atlas of eukaryotic genes. Nature Communications 9:373.
6. Zayed AA, Wainaina JM, Dominguez-Huerta G, Pelletier E, Guo J, Mohssen M, Tian F, Pratama AA, Bolduc B, Zablocki O, Cronin D, Solden L, Delage E, Alberti A, Aury JM, Carradec Q, da Silva C, Labadie K, Poulain J, Ruscheweyh HJ, Salazar G, Shatoff E,

Bundschuh R, Fredrick K, Kubatko LS, Chaffron S, Culley AI, Sunagawa S, Kuhn JH, Wincker P, Sullivan MB. 2022. Cryptic and abundant marine viruses at the evolutionary origins of Earth's RNA virome. Science 376:156–162.

7. Rittmann BE, McCarty PL. 2001. Environmental Biotechnology: Principles and Applications. McGraw-Hill: New York, NY.

8. Redfield AC. 1958. The biological control of chemical factors in the environment. American Scientist 46:205–221.

9. Thompson PA, Harrison PJ, Parslow JS. 1991. Influence of irradiance on cell volume and carbon quota for ten species of marine phytoplankton. Journal of Phycology 27:351–360.

10. Healey FP. 1985. Interacting effects of light and nutrient limitation on the growth rate of *Synechococcus linearis* (Cyanophyceae). Journal of Phycology 21:134–146.

11. Chalup MS, Laws EA. 1990. A test of the assumptions and predictions of recent microalgal growth models with the marine phytoplankter *Pavlova lutheri*. Limnology and Oceanography 35:583–596.

12. Menden-Deuer S, Lessard EJ. 2000. Carbon to volume relationships for dinoflagellates, diatoms, and other protist plankton. Limnology and Oceanography 45:569–579.

13. Claquin P, Martin-Jézéquel V, Kromkamp JC, Veldhuis MJW, Kraay GW. 2002. Uncoupling of silicon compared with carbon and nitrogen metabolisms and the role of the cell cycle in continuous cultures of *Thalassiosira pseudonana* (Bacillariophyceae) under light, nitrogen, and phosphorus control. Journal of Phycology 38:922–930.

14. Werner D. 1977. Silicate metabolism. In Werner. D. [Ed.] The Biology of the Diatoms. University of California Press: Berkeley, CA.
